# Research Progress on Leucine-Rich Alpha-2 Glycoprotein 1: A Review

**DOI:** 10.3389/fphar.2021.809225

**Published:** 2022-01-05

**Authors:** Yonghui Zou, Yi Xu, Xiaofeng Chen, Yaoqi Wu, Longsheng Fu, Yanni Lv

**Affiliations:** ^1^ Department of Pharmacy, The First Affiliated Hospital of Nanchang University, Nanchang, China; ^2^ School of Clinical Medicine, Nanchang University, Nanchang, China; ^3^ College of Pharmacy, Nanchang University, Nanchang, China

**Keywords:** LRG1, review, TGF- beta 1, nervous, tumor

## Abstract

Leucine-rich alpha⁃2 glycoprotein 1 (LRG1) is an important member of the leucine-rich repetitive sequence protein family. LRG1 was mainly involved in normal physiological activities of the nervous system, such as synapse formation, synapse growth, the development of nerve processes, neurotransmitter transfer and release, and cell adhesion molecules or ligand-binding proteins. Also, LRG1 affected the development of respiratory diseases, hematological diseases, endocrine diseases, tumor diseases, eye diseases, cardiovascular diseases, rheumatic immune diseases, infectious diseases, etc. LRG1 was a newly discovered important upstream signaling molecule of transforming growth factor⁃β (TGF⁃β) that affected various pathological processes through the TGF⁃β signaling pathway. However, research on LRG1 and its involvement in the occurrence and development of diseases was still in its infancy and the current studies were mainly focused on proteomic detection and basic animal experimental reports. We could reasonably predict that LRG1 might act as a new direction and strategy for the treatment of many diseases.

## Introduction

**FIGURE 1:**
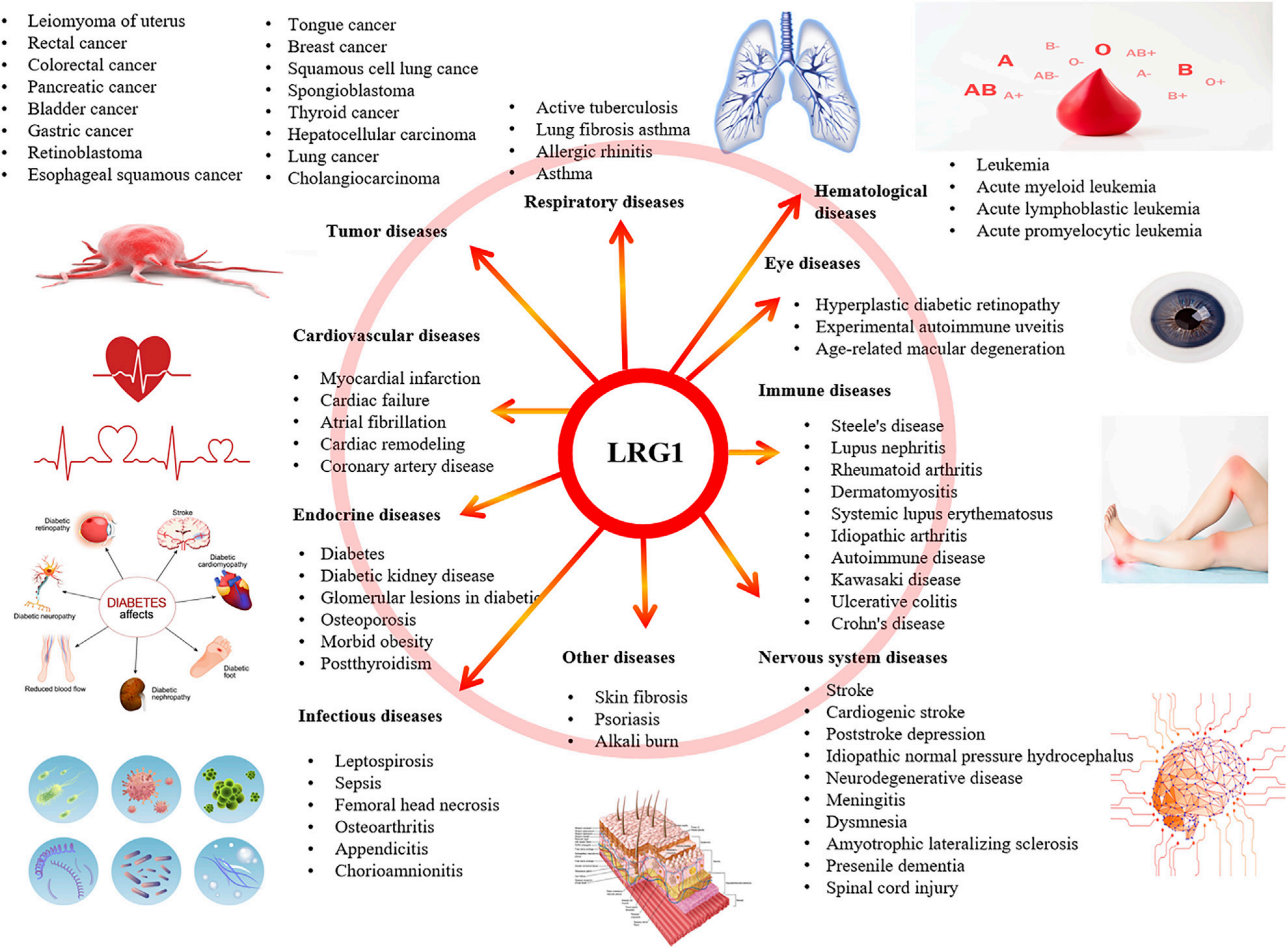
LRG1 involved in the related diseases.

The incidence of refractory chronic diseases is increasing year by year, and this is always a challenging aspect of medical work. Increasing numbers of studies have found that leucine α⁃2 glycoprotein⁃1 (LRG1) engaged in signal transduction and pathogenensis of multiple diseases. Leucine-rich α2-glycoprotein 1 (LRG1), first separated from human serum in 1977, is a family member of the leucine-rich repeating family, consisting of eight leucine-rich repeats (mostly 20–30 amino acid residues in length) (Liu et al., 2017). The LRG1 gene located on the short arm of chromosome 19, band 3, and region 13 (19P13.3). The mature form of LRG1 is a secreted protein isolated from human serum (Haupth and Baudner, 1977), while its amino acid sequence was determined in 1985 with 312 amino acids (Takahashi et al., 1985). The molecular weight of LRG1 is 45 kD, and its equipotential point is 4.52–4.72. The initial studies have demonstrated that most leucine-rich repeat proteins were detected with high expression as transmembrane proteins in the central nervous system, which mainly played role in the normal physiological activities of the nervous system, such as synapse formation, synapse growth, the development of nerve processes, neurotransmitter transfer and release, and cell adhesion molecules or ligand binding proteins. The expression of LRG1 could be detected in disease specimens, and LRG1 expression had also been detected in the blood of patients, and it had relatively stable specificity and sensitivity. LRG1 could act as a new biological marker for inflammatory diseases and some tumors, which was very important for the diagnosis and prognostication of diseases (Figure 1). It has been reported that LRG1 mainly exerted its functions through the transforming growth factor⁃β (TGF⁃β) pathway. This review expected to describe the full implication of the LRG1 in related diseases.

### Part One: LRG1 Involved in Respiratory Diseases

Findings in clinical respiratory diseases suggested that LRG1 was a promising biomarker for the detection of active tuberculosis, lung fibrosis, asthma, allergic rhinitis, and asthma. LRG1 serum levels in tuberculosis patients were significantly higher than those in healthy controls group, and decreased after 1 month of anti-tubercular therapy (Fujimoto et al., 2020). Inhibitory actions of allergens responsible for allergic airway disorders might reduce levels of LRG1 and transforming growth factor beta receptor II in patients.(Hao L. et al., 2016). In basic research related with respiratory related diseases, LRG1 promoted lung fibrosis by regulating the phosphorylation of TGF-β and smad2 in the profibrotic activation of fibroblast (Honda et al., 2017). LRG1 was highly expressed in a subpopulation of bronchial epithelial cells in asthma model mice (Honda et al., 2016). A pathway that inhibition of LRG1 might rescue vascular rarefaction and alveolar regression for the treatment of chronic obstructive pulmonary disease or emphysema (Hisata et al., 2021).

### Part Two: LRG1 Involved in Hematological Diseases

LRG1 was found to be important in hematological diseases, and the association was primarily with leukemia. Bioinformatics analysis revealed that some proteins, namely, LRG1, S100A8, SPARC (secreted protein acidic and rich in cysteine), and sL-selectin (human recombinant soluble L-selectin) were closely related to childhood T-cell acute lymphoblastic leukemia and acute promyelocytic leukemia (Yu R. et al., 2019;Yu, Zhang, et al., 2021). On hematological diseases cellular model, healthy hematopoietic stem progenitor cells overexpressed the leukemia stem cell-specific genes LY86, LRG1, and PDE9A under the induction of leukemia microvesicles (Razmkhah et al., 2019). The silencing of LRG1 could reduce viability and promote apoptosis in leukemia KASUMI-1 cells through regulating cyclin and apoptosis-related proteins (Xiao and Zhu, 2018). In addition, alpha-2-HS-glycoprotein and LRG1 were the most prospective candidates for different myelodysplastic syndrome subgroups, including refractory cytopenia with multilineage dysplasia, refractory anemia or refractory anemia with ringed sideroblasts, etc (Majek et al., 2015).

### Part Three: LRG1 Involved in Endocrine Diseases

LRG1 could be further excavated as a potential target for the pathological progress of diabetic kidney disease, including glomerular lesions in diabetes (Fu et al., 2018; Liu J.-J. et al., 2021; Iqbal et al., 2021), lysosomal function related Type 1 diabetes (Singh et al., 2017), and nephropathy in type 2 diabetes. (Liu et al., 2017; Gurung et al., 2021). Among Asian patients with type 2 diabetes, low baseline skeletal muscle mass was tightly related with the growing risk of chronic kidney disease progression. Pigment epithelial-derived factor and LRG1 mediated a negative association between skeletal muscle mass index and chronic kidney disease in Asians with progression to type 2 diabetes (Low et al., 2021). Knockdown of LRG1 might significantly ameliorate diabetes-induced diabetic glomerulopathy, podocyte loss, and glomerular angiogenesis, while its mechanism was connected with the activation of ALK1 (activin receptor-like kinase)-smad1/5/8 in the glomeruli of diabetic mice (Hong et al., 2019). Glomerular LRG1 expression was enhanced in db/db mice, accompanied by higher expressed glomerular fibrosis associated genes compared with that in nondiabetic db/m mice (Haku et al., 2018).

In addition, patients with diabetes are at high risk of complications from cardiac, cancer, and angiogenesis outcomes. Plasma LRG1 might potentially be involved in the pathogenesis of heart failure in type 2 diabetes patiens (Liu J. J. et al., 2021). Several biomarkers consisting of apolipoprotein A-IV, monocyte differentiation antigen CD14, and LRG1 demonstrated their influences on the differentiation between diabetes and early pancreatic cancer (Peng et al., 2020). LRG1 was a novel proangiogenic factor involved in abnormal angiogenesis and renal fibrosis in diabetic nephropathy (Zhang A. et al., 2020). LRG1 promoted wound repair and regenerate nerves of diabetic corneal epithelium by regulating matrix metalloproteins (Li et al., 2020).

Furthermore, LRG1 was involved in other endocrine diseases and has key capabilities for the pathological process of diseases. LRG1 could be applied as a biomarker to detect glomerular damage in kidney disease (Lee et al., 2018; Jiang et al., 2020). Serum LRG1 participated in the prediction of the mortality of cardiovascular morbidity complication in end-stage renal disease patients (Yang FJ. et al., 2020). Weight loss after metabolic/bariatric surgery was connected with low level of plasma LRG1, suggesting it is a potential biomarker of inflammation and obesity (Pek et al., 2018). The increased proteins of haptoglobin, hemopexin, and LRG1 were found from hyperthyroidism patients with the treatment of antithyroid (Masood et al., 2020). LRG1 downregulation promoted osteoblast viability and collagen synthesis by activating the TGF-β/smad signaling pathway (Gu et al., 2020).

### Part Four: LRG1 Involved in Tumor Diseases

Tumors are serious diseases endangering people’s lives, and their early diagnosis and targeted treatment have become hotspots of research (Chen et al., 2016). Reports on the relationship between intestinal cancer and LRG1 have rapidly increased in the last 5 years. LRG1 response to preoperative chemoradiotherapy might help in prognostication and the selection of treatments after preoperative chemoradiotherapy (Lee et al., 2019). LRG1 with fucosylated triantennary N-glycan were detected as a new combined colorectal cancer marker, which had higher sensitivity exceeding CA19-9 (Shinozaki et al., 2018). Additionally, LRG1 served as a biomarker to detect early high-risk adenomas as well as colorectal cancer (Sun et al., 2017; Komor et al., 2020; Kopylov et al., 2020). significantly decreased combined indexes of stem cell factor, LRG1, and platelet lymphocyte ratio could be detected in nontreated colorectal cancer patients (Fouda et al., 2021). Basic research results suggested that LRG1 promoted the cellular proliferation and apoptosis by modulating runt-related transcription factor 1 expression in colorectal cancer (Zhou et al., 2017). Extracellular vesicles derived from serum in colon cancer might promote metastasis through the modulating of extracellular matrix-related proteins, secreted protein acidic and rich in cysteine, and LRG1 (Zhong et al., 2019). Long noncoding RNA prostate miR-150-5p/LRG1 pathway facilitated the malignant progression of colorectal cancer and might provide a targeted colorectal cancer therapy (Lou et al., 2020). LRG1 could promote the cellular invasion and growth, which might act as the outcomes of colorectal cancer patients (Zhang et al., 2018).

LRG1 was identified as a biomarker for tumors of the digestive system. In addition to intestinal cancer, LRG1 could act as the biomarker for esophagus cancer, gastric carcinoma, and bile duct cancer, etc. Overexpression of LRG1 negatively regulated TGF-β signaling pathways to inhibite cellular migration and invasion of esophageal squamous (Zhang N. et al., 2020). LRG1 was an independent indicator of poor clinical outcomes in esophageal squamous cell carcinoma (Wang et al., 2019). Combound biomarker of LRG1 and C-reactive protein and soluble interleukin-6 receptor could forecast the response to preoperative chemoradiotherapy in esophageal cancer patients (Nambu et al., 2019). Serum LRG1 of gastric cancer patients was significantly higher than that of healthy volunteers, and increased with the progression of gastric cancer pathological stage (Yamamoto et al., 2017). The upregulation of LRG1 associated with TGF-β1 expression served as an independent factor for patients with postoperative intrahepatic cholangiocarcinoma (Jin et al., 2020). A metabolite panel in combination with CA19-9, tissue inhibitor of metalloproteinase 1, and LRG1 exhibited substantially improved performance in the detection of early-stage pancreatic ductal adenocarcinoma (Fahrmann et al., 2019). Prediagnostic cases below the cutoff value for CA19-9, in combination with LRG1 and TIMP1, was identified as useful for the early detection of pancreatic cancer (Choi et al., 2021; Fahrmann et al., 2021); also, these results were strongly supported in a pancreatic cancer animal model (Fukamachi et al., 2019; Harlid, Gunter, and Van Guelpen 2021; Lee et al., 2021). Molecular mechanistic research hinted that LRG1 strong enhance the cellular migration and invasion of pancreatic ductal adenocarcinoma cells *in vitro* through activation of the p38/MAPK signaling pathways (Xie ZB. et al., 2019).

Outside tumors of the digestive system, LRG1 was found to be the biomarker in other tumor diseases. For isocitrate dehydrogenase one wild-type glioblastoma, high expression of LRG1 was regarded as an independent factor (Furuta et al., 2020). The plasma concentrations of LRG1, C-reactive protein, and complement component C9 showed significant positive correlations with tumor size in glioblastoma patients (Miyauchi et al., 2018). MiR-335 regulated LRG1 and positively suppressed the invasion of neuroblastoma cells (Lynch et al., 2012). Elevation expression of LRG1 and decreased Ki-67 were showed by SET domain-containing 1A histone lysine methyltransferase knockdown. Enhanced expression of LRG1 in Hs578T might distinguish poor outcomes from triple-negative breast cancer (Mohamad Hanif 2019). Abnormal high expression of LRG1 levels promoted lymphatic metastasis and cellular apoptosis in the malignant progression of breast cancer (Zhang YS. et al., 2021; Jemmerson et al., 2021). LRG1 alone or in combination with CA125 might be a stool biomarker in the diagnosis of epithelial ovaries (Wu et al., 2015).

Recent advances in the interpretation of the molecular mechanism of LRG1 in tumor indicated that long noncoding RNA taurine upregulated one mediated endothelial angiogenesis through the LRG1/TGF-β pathway (Fan et al., 2019), which could predict clinical outcomes after transarterial chemoembolization (Peng et al., 2020). Elevated serum levels of TGF-β1 and LRG1 were associated with morbidity and severity of uterine leiomyoma (Kamalipooya et al., 2021). Other findings imply that LRG1 was correlated with the diagnosis of squamous cell carcinoma of the head and neck (Wang et al., 2017a), tongue carcinoma (Hao L. J. et al., 2016), oral cancer (Chang et al., 2019), retinoblastoma (Luan et al., 2021), thyroid carcinoma (Ban et al., 2019), hepatocellular carcinoma (Yu et al., 2017), lung adenocarcinoma (Pu et al., 2020), and diffuse large B-cell lymphoma (Yu J. et al., 2019). Non-small-cell lung cancer or renal cell carcinoma induced an enhancement of cell proliferation, migration, and invasion via the LRG1-mediated TGF-β pathway (Chokchaichamnankit et al., 2019; Li et al., 2019; Hong et al., 2020).

### Part Five: LRG1 Involved in Eye Diseases

LRG1 not only showed relevant mechanisms associated with diabetes but also diabetes-related eye diseases. Baseline plasma LRG1 was associated with proliferative diabetic retinopathy, suggesting that it might be a desirable biomarker to predict the late proliferative stage of diabetic retinopathy (Zhang et al., 2019). LRG1 levels in plasma and vitreous were increased in the individuals with proliferative diabetic retinopathy (Chen C. et al., 2019). LRG1 had applications for other eye diseases. Protein expression of five genes, serpina3n (serine protease inhibitor A3N), lcn2 (lipocalin-2), ackr1 (atypical chemokine receptor 1), LRG1, and lamc3 (laminin subunit gamma 3), were validated at the level of the inner blood retinal barrier cells (Lipski et al., 2020). The level of LRG1 obtained from eyes in patients with neovascular age-related macular degeneration were increased, which indicated the target therapeutic therapy of anti-LRG1 monoclonal antibody (Mundo et al., 2021). LRG1 activated NADPH oxidase four to promote the epithelial mesenchymal transition of retinal pigment epithelium cells, which explored the potential mechanism for subretinal fibrosis in the basic experiment (Zhou et al., 2021).

### Part Six: LRG1 Involved in Nervous System Diseases

There were many etiologies that could cause neurological diseases, including poisoning, genetic defects, nutritional disorders, immune damage, metabolic disorders, and endocrine disorders. There were relatively comprehensive clinical and basic studies on the function of LRG1 in stroke diseases. Serum level of LRG1 was identified as a potential indicator for the prediction of cardioembolic stroke, as well as pediatric spinal cord injury (Zhang M. et al., 2021; Ma et al., 2021). Apolipoprotein CII, LRG1, and C-reactive protein expression were significantly downregulated in poststroke depression relative to stroke subjects (Zhan et al., 2014). On fundamental researches, microglial M2 polarization was marked with chitinase 3-like protein one and LRG1 in bone marrow-resident monocytes induced by ischemia (Gregory et al., 2010). LRG1 might promote the cascade of angiogenesis via the modulation of TGF-β1 pathway in middle cerebral artery occlusion rat. The glucose concentration affected LRG1 methylation in cortical slices and could modify neurodevelopmental outcomes (Chong et al., 2018) LRG1 promoted apoptosis and autophagy via the regulation of the TGF-β-smad1/5 signaling pathway by upregulating activin receptor-like kinase 1, which exacerbated ischemia reperfusion injury (Jin et al., 2019). Blockade of LRG1 attenuated angiogenesis by mediating its modulation of TGF-β signaling (Wang et al., 2013).

So far, the etiology of many neurological diseases was unknown, and new biomarkers for nervous system diseases should be identified. A combination of positive tests such as LRG1 and tau protein could reliably predict the outcome in elderly patients with Idiopathic atmospheric hydrocephalus (Nakajima, Arai, and Miyajima 2010; Nakajima et al., 2011). An abonormal accumulation of LRG1 in brain of parkinson patients with subtypes of dementia and progressive supranuclear palsy was deemed to be a cause of neurodegeneration (Miyajima et al., 2013). Additionally, LRG1 shaped hippocampal circuits and establishes the assembly of tyrosine kinase receptor B, with LRG1 expanding the repertoire of responses to brain-derived neurotrophic factors during brain development (Alsina et al., 2016). LRG1 got more superior performance than interleukin-6 in the prediction for inflammatory diseases of the central nervous system (Chong et al., 2018). Cartier et al. (2018) Hippocampal LRG1 overexpression contributed to fewer synaptic vesicles and junctions, which led to memory impairment with increased age (Akiba et al., 2017). Three protein expression LRG1, secretoglobin family 3A member 1, and peptidoglycan recognition protein one were mainly associated with inflammation and apoptosis and they regulated the regeneration of nerves (Zheng et al., 2019). The perilipin 4, lipocalin-2, LRG1, forkhead Box F1, and cytotoxic T lymphocyte-associated protein two alpha genes were significantly upregulated in sevoflurane-induced Alzheimer’s-related neuropathology in mice (Ge et al., 2019). LRG1 expression increased in resident astrocytes with age (Nakajima et al., 2012).

### Part Seven: LRG1 Involved in Cardiovascular Diseases

LRG1 has promising application value in clinical diagnosis and prediction for congestive heart failure and cardiomyopathy (Liu M. et al., 2020). Omics results suggested that metabonomics was a promising new biomarker for cardiovascular diseases. LRG1 might be a potential serum biomarker for early onset myocardial infarction, while a combined biomarker signature that included BNP (plasma brain natriuretic peptide) would be a more accurate predictor of heart failure than BNP alone (Xuan et al., 2019). TLRG1 was the most prominent biomarker, among others, to predict the pathological progress of heart failure (Tonry et al., 2021). LRG1 was selected to distinguish persistent atrial fibrillation patients and control subjects (Cao et al., 2020). Predictive molecules of LRG1 and miRNAs engaged in pathological development of stable coronary artery disease progressing to acute myocardial infarction (Xiao et al., 2021). Proteomic study of serum exosomes from patients with kawasaki disease coronary aneurysm identified four proteins, TN, RBP4, LRG1, and APOA4 as the specific biomarkers (Xie XF. et al., 2019). Due to their great potential in the prediction of cardiovascular diseases, particular methods and mechanisms had yet to be investigated. Uncovering potential lncRNAs and mRNAs, including LRG1 was essential biomarkers from acute myocardial infarction aggravated to heart failure (Tonry et al., 2021; Wang et al., 2021). In terms of mechanism research, overexpressed miR-494 bound with LRG1 inactivated Wnt signaling pathway might promote fibroblasts and vascular endothelial cells proliferation in myocardial infarction (Su et al., 2019). LRG1 or PPARβ/δ represented a promising therapeutic strategy for the inhibition of pathological cardiac reshaping (Liu et al., 2019).

### Part Eight: LRG1 Involved in Immune Diseases

The cause of the immune disease is complicated, and there is little cure for its problems. Generally, not only the initiator but also a series of inflammatory introduction systems activated on the basis of immunity could be investigated to determine the causes of rheumatoid arthritis. Plasma levels of LRG1 were relevant with disease activity of lupus nephritis (Yang Y. et al., 2020). Urine levels of LRG1, orosomucoid one ans two were positively associated with diseases of urinary system (Sun et al., 2020). LRG1 specially expressed in the acute phase of kawasaki disease rather than in the convalescence of Kawasaki disease (Kimura et al., 2017; Yanagimachi et al., 2021). The serum level of LRG1 could validly predict progression and prognosis of interstitial pneumonia with dermatomyositis (Ishida et al., 2020). Serum LRG1 levels were normalized in the inactive systemic juvenile idiopathic arthritis phase after treatment (Shimizu et al., 2019). During the treatment of interleukin-6 receptor blockers, serum levels of LRG1 has good prediction ability for systemic juvenile idiopathic arthritis (Shimizu et al., 2017). Serum LRG exerted its superiority to mucosal healing in ulcerative colitis rather than C-reactive protein levels (Shinzaki et al., 2017). Serum LRG1 could also act as a useful biomarker to monitoring disease activity of inflammatory bowel disease during anti-TNF treatment (Shinzaki et al., 2021).

In basic research related to immunity diseases, interleukin-22 also promoted the expression of ERK1/2 (extracellular signal regulated kinase)-independent genes, such as LRG1, which were involved in inducing cell proliferation in intestinal epithelial cells (Moniruzzaman et al., 2019). PPARβ/δ regulated LRG1 in fibroblasts through TGF-β1 could promote the pathological progression of skin diseases (Sng et al., 2018). Inhibition of TNF-α (tumor necrosis factor α) and LRG1 by lenalidomide by induction of angiogenic cascade and recruitment of mesenchymal stem cells in subchondral bone could be a potential therapeutic approach for *de novo* bone formation (Wang et al., 2017b). LRG1 facilitated the activities of Th17 differentiation related arthritis diseases via upregulating interleukin-6 expression in Naive CD4 T cells (Urushima et al., 2017).

### Part Nine: LRG1 Involved in Infectious Diseases

Infections could be distributed in any part of the body, which has always attracted attention from the public. There were significant differences in expression levels of LRG1 and α-1-antichymotrypsin between the healthy group and the leptospirosis group, but were no significant differences in the dengue control group (Fish-Low et al., 2020). A five protein panel of complement factor H related 5, LRG1, C-reactive protein, lipopolysaccharide binding protein, and serum amyloid A1 had specific predictive power in distinguishing tuberculosis and other respiratory diseases (Garay-Baquero et al., 2020). The ROC (receiver operating characteristic curve) identified nine transcriptomic genes, including LRG1, as potential new biomarkers for sepsis (Gong et al., 2020). Ten genes included LRG1 were differentially expressed in sepsis compared with nonsepsis blood (Lu et al., 2020) (Hashida et al., 2017). In the basic study of infectious diseases, LRG1 participated in FOS-like 1-regulated gene expression during lipopolysaccharide-induced human lung pulmonary endothelial cell angiogenesis (Nitkin et al., 2020). LRG1 repressed cellular signal-regulated kinase one activity by downregulating GTPase cell division cycle 42, and its downstream mitogen-activated protein kinase cascade diminished fungal virulence in a mouse model of *Candida albicans* infection (Chen T. et al., 2019).

LRG1 provided a novel biomarker for the evolution of cellular microbiology and infectious diseases. LRG1 stimulated combined with inflammatory cytokines served as a promising biomarker for infection of fetal hepatocytes (Kajimoto et al., 2020). Plasma proteomics of the kidney tissue obtained from newborn lippolysaccharide pigs showed elevated five protein levels involving LRG1, which were associated with activation of natural immunity (Muk et al., 2020). Circulating human mRNA and protein levels of LRG1 had good prediction ability for acute appendicitis in adults or children (Rainer et al., 2017; Kakar et al., 2021). Incorporated urinary biomarker model with LRG1, constant pain, right iliac fossa tenderness, and pain on percussion play the critical role in the prediction for appendicitis in children (Yap et al., 2019). However, there were also studies that showed that plasma levels of LRG1 were ineffective in the diagnosis of acute appendicitis in female patients accompanied by acute abdominal pain (Demirci et al., 2017). In addition, LRG1 and other five genes were found to be the key genes in steroid-induced femoral head necrosis. TNF-α-induced LRG1 secretion could recruit MSCs to osteoarthritic subchondral bones via promoting vessel regeneration coupled with new bone formation (Verweyen et al., 2021; Yang et al., 2021).

### Part Ten: LRG1 Involved in Other Diseases

LRG1 was an innovative biomarker for psoriasis, immunoglobulin G4-related disease, and fibromyalgia (Nakajima et al., 2017; Hsu et al., 2021; Kawanami et al., 2021). LRG1 combined with other five proteins could act as the biomarkers well connected with middle or end coronary events in atherosclerotic patients with any genetic subtypes (Bos et al., 2017). Additionally, in some basic research experiments, LRG1 might uncouple mechanical forces necessary for angiogenesis, which underline the potential therapeutic methods for fibro-proliferative diseases (Gao et al., 2019). Mesenchymal stem-derived extracellular vesicles microrNA-129-5p could attenuate the degeneration of intervertebral disc mediated by LRG1 inactivated P38 MAPK signaling pathway (Yang et al., 2021). Knockdown of LRG1 inhibited corneal angiogenesis and lymphangiogenesis in corneal alkali burn mice by modulating the protein expression of vascular endothelial growth factor A, B, C, D (Song et al., 2020). LRG1 stimulated neutrophil infiltration by regulating IL-6/Stat3 (signal transducer and activator of transcription 3) to facilitate the corneal fibrosis responses (Yu, Yang, et al., 2021).

## Discussion

At present, many proteins with leucine-rich repeat structures had been found in the nervous system, other systems, and body fluids, and research on their functions was still a hot research direction in the future. In recent years, an increasing number of studies have found that LRG1 could exert its effects by promoting the TGF⁃β signaling pathway and then affecting downstream biological effects through smad-dependent and non-smad-dependent pathways. In mammals, TGF-β mainly was identified with three subtypes: TGF-β1, TGF-β2, and TGF-β3, among which TGF-β1 exerted a large portion of its biological effects.

**FIGURE 2:**
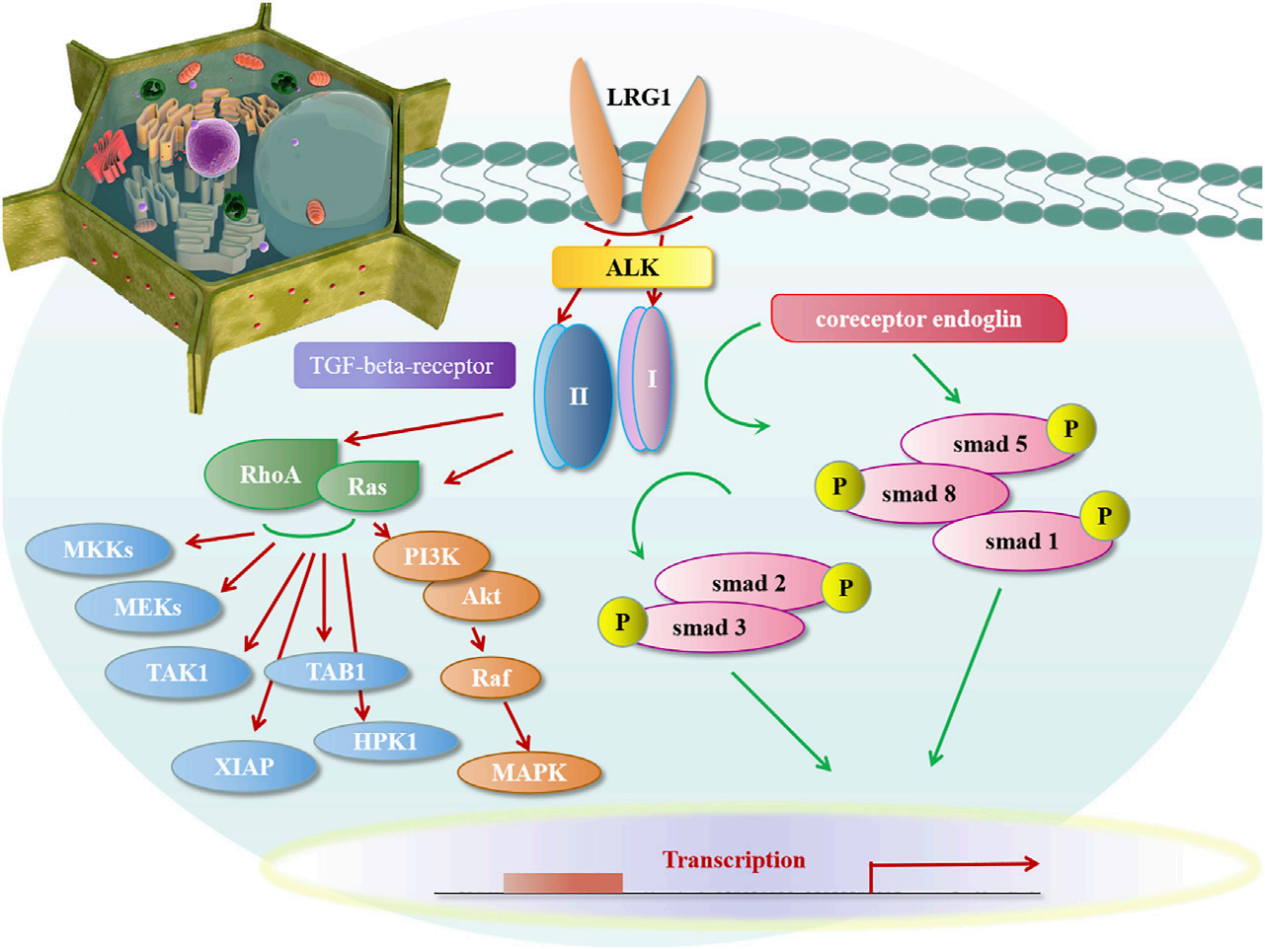
LRG1 activated TGF-β signaling pathway.

During this process, LRG1 played an important role in the TGF⁃β1 pathway: 1) (Figure 2) Endoglin was proposed as a key regulatory molecule in promoting signalling through the ALK (activin receptor-like kinase) pathway. In the presence of coreceptor endoglin, LRG1 activated the TGF angiogenic switch binding to the accessory receptor endoglin by in the presence of TGF-β1. The activation of TGF-β1 bound with TGF-beta-receptor-II, which predominantly recruited the endothelial TGF-beta-receptor-I and ALK to the highly conserved proximal membrane region to undergo phosphorylation. Then the phosphorylation of TGF-beta-receptor-I/ALK stimulated Smad receptor family to form the hetero-oligomic complex and transport to the nucleus, and then interacted with transcriptional coactivators and co-inhibitors, such as P300 and cAMP-response element binding protein to mediate the biological effects of TGF-β1. The complex of LRG1-TGF-beta-receptor-I-endoglin-ALK1 activated the downstream smad1/5/8 pathway and promoted endothelial cell migration and angiogenesis (Wang et al., 2013); 2) ALK1 and ALK5 were the both direct receptors of TGF-β1, through antagonizing, supplementing, and restricting each other to maintain body balance. Inparticular, the balance between the ALK5 and ALK1 signalling pathways is considered to becentral in determining the angiogenic switch. Under the influence of different factors, they showed different tendencies towards TGF-β1. In the absence of coreceptor endoglin, LRG1 would combine with TGF-beta-receptor-I/ALK5 to form a complex of LRG1⁃TGF-beta-receptor-I/ALK5, which stimulated the downstream smad2/3 pathway and promoted the deposition of extracellular cytoplasm. It regulated the differentiation of T cells and promoted the synthesis of endothelial nitric oxide synthase (Wang et al., 2013). 3) In addition to this, LRG1-induced TGF⁃β pathway could activate upstream signaling molecules RhoA, Ras to activate MKKs (MAP kinase), MEKs (MAPK/ERK kinase), mitogen-activated protein kinase (JNK/SPAK, p38, ERK1/2), TAK1 (TGF-Beta Activated kinase), TAB1 (TAK1 Binding Protein), XIAP (*Xenopus* Inhibitor of Apoptosis), HPK1 (Haematopoietic Progenitor Kinase-1), PI3K (phosphatidylinositol-3-kinase), and AKT kinase, etc (Moustakas et al., 2002; Travis and Sheppard, 2014; Costanza et al., 2017; Liu C. et al., 2020).

## Conclusion

In conclusion, it is particularly important to explore new therapeutic drugs and schemes to reduce the economic burden of patients and the development of diseases. LRG1 was a newly discovered important upstream signaling molecule of TGF-β that affects various pathological processes through the TGF-β signaling pathway. Although research on LRG1 in the occurrence and development of diseases was still in its infancy, clinical evidence was still scarce, mainly concentrated on proteomic detection and basic experimental reports. However, the monoclonal antibody magacizumab specifically targeting LRG1 was currently in phase I and II clinical trials (Radgonde et al., 2018). We could reasonably predict that LRG1 might serve as a novel target for treatment of disease disorders.
